# Acute Dystonia Secondary to Domperidone in a Pediatric Patient

**DOI:** 10.7759/cureus.3587

**Published:** 2018-11-13

**Authors:** Amir Shahbaz, Karam Elahi, Muhammad Affan, Muhammad Faizan Shahid, Ahtesham Sabir

**Affiliations:** 1 Internal Medicine, Icahn School of Medicine at Mount Sinai/Queens Hospital Center, New York, USA; 2 Internal Medicine, Punjab Medical College, Allied Hospital, Faisalabad, PAK; 3 Internal Medicine, King Edward Medical University, Lahore, PAK; 4 Internal Medicine, Jinnah Hospital/Allama Iqbal Medical College, Lahore, PAK; 5 Internal Medicine, Ayub Medical College, Abbottabad, PAK

**Keywords:** domperidone, dystonia

## Abstract

Domperidone is a dopamine-2 receptor (D2) antagonist. It is used as an antiemetic and has an excellent safety profile. We present a case of acute dystonia secondary to domperidone use in an 11-year-old girl. She was admitted with the diagnosis of viral gastroenteritis and received domperidone for persistent vomiting along with other supportive measures. The vomiting subsided with treatment, but she developed acute dystonia. Domperidone discontinued, and her condition improved in the next 24 hours. The patient discharged, and on a follow-up visit, she was asymptotic. A review of the literature suggests a possible association of acute dystonia with domperidone in children.

## Introduction

Domperidone is a peripheral dopamine-2 receptor (D2) antagonist and used as a prokinetic and antiemetic drug. It acts on the chemoreceptor trigger zone and motor functions of the stomach and small intestine. It has negligible penetration through the blood-brain barrier and provides an excellent safety profile in the recommended doses [1]. Rarely, in children, acute extrapyramidal symptoms due to domperidone use observed. The most likely mechanism is the underdeveloped blood-brain barrier [2].

## Case presentation

An 11-year-old girl presented with fever, body aches, diarrhea, and persistent vomiting for two days. She was conscious and alert. Her heart rate was 92/min, blood pressure 115/70 mmHg, and temperature 39.5 °C. There was no pallor, icterus, cyanosis, pedal edema, or lymphadenopathy. The cardiovascular, respiratory, neurological, and abdominal examination was unremarkable. Blood work showed hemoglobin 11.4 g/dl, total leucocyte count 10,200 cell/mm, differential leukocyte count (DLC) 41%, lymphocytes 56%, monocytes 2%, and eosinophils 1%. Liver function tests (LFT), urea creatinine, random blood sugar, and urine analysis were within the normal range.

Diagnosis of an acute viral gastroenteritis was made, and she received symptomatic treatment with intravenous acetaminophen and domperidone. On the second day of admission, she was afebrile, and vomiting stopped. However, she developed involuntary spastic arching of the back and spontaneous involuntary movements of the lips and tongue. Neurology consultation was sought, and after a detailed neurological examination, domperidone-induced acute dystonia was proposed as a probable diagnosis. There was no history of head injury or epilepsy. Rest of the physical examination was unremarkable. LFT, urea creatinine, and urinalysis were repeated and yielded normal results. Domperidone was discontinued immediately and promethazine was prescribed. Within 24 hours, her condition improved and abnormal movements disappeared. The patient was discharged, and during the four-week and six-month follow-up visits, no recurrence was observed.

## Discussion

Domperidone is a peripheral D2 antagonist used for the treatment of vomiting, gastroparesis, and hiccoughs [3]. Its common side effects are stomach cramps, diarrhea, and constipation. Cardiac arrhythmias are rare but may cause sudden death [4]. Extrapyramidal symptoms such as dystonias, Parkinsonism, akathisia, and tardive dyskinesia are side effects of centrally acting D2 antagonists, such as antipsychotics. Rarely, these side effects are observed with domperidone use, especially in infants and young children due to the poorly developed blood-brain barrier [5]. Drug-induced dystonia may be confused with different conditions such as partial seizure, encephalitis, tetany, tetanus, and electrolyte imbalances. Drug-induced acute dystonia is a diagnosis of exclusion [6-7].

Careful physical examination and history, especially of the drug intake in the recent past, are essential. The temporal association between the administration of domperidone and the onset of acute dystonia and the resolution of symptoms with the discontinuation of the drug suggests that domperidone is the most likely culprit. A detailed review of this patient's case, using the Naranjo probability scale, shows a probable association between domperidone and acute dystonia [8]. Domperidone-induced acute dystonias are reversible and treated with parenteral administration of anticholinergics such as benztropine or diphenhydramine. Antihistamines having an anticholinergic action such as promethazine are also helpful [5,9].

## Conclusions

Extrapyramidal symptoms such as acute dystonia are rare side effects of domperidone, especially in the pediatric population, and manifest as an involuntary contraction of various muscles. When a child, as in our case, receiving domperidone, develops abnormal involuntary movements, drug-induced acute dystonia should be considered as a probable diagnosis after ruling out other potential contributing factors.
